# An Inducible and Vascular Smooth Muscle Cell-Specific Pink1 Knockout Induces Mitochondrial Energetic Dysfunction during Atherogenesis

**DOI:** 10.3390/ijms22189993

**Published:** 2021-09-16

**Authors:** Craig K. Docherty, Jordan Bresciani, Andy Carswell, Amrita Chanderseka, Elaine Friel, Marianna Stasi, John R. Mercer

**Affiliations:** Institute of Cardiovascular and Medical Sciences, College of Medical Veterinary and Life Sciences, University Avenue, University of Glasgow, Glasgow G12 8TA, UK; craig.docherty@glasgow.ac.uk (C.K.D.); jordan.bresciani@glasgow.ac.uk (J.B.); andy.carswell@glasgow.ac.uk (A.C.); amrita.chanderseka@glasgow.ac.uk (A.C.); elaine.friel@glasgow.ac.uk (E.F.); marianna.stasi@glasgow.ac.uk (M.S.)

**Keywords:** mitochondrial dysfunction, atherosclerosis, DNA damage, oxidative phosphorylation, glycolysis

## Abstract

DNA damage and mitochondrial dysfunction are defining characteristics of aged vascular smooth muscle cells (VSMCs) found in atherosclerosis. Pink1 kinase regulates mitochondrial homeostasis and recycles dysfunctional organelles critical for maintaining energetic homeostasis. Here, we generated a new vascular-specific Pink1 knockout and assessed its effect on VSMC-dependent atherogenesis *in vivo* and VSMC energetic metabolism *in vitro*. A smooth muscle cell-specific and MHC-Cre-inducible flox’d Pink1^f/f^ kinase knockout was made on a ROSA26^+/0^ and ApoE^−/−^ C57Blk6/J background. Mice were high fat fed for 10 weeks and vasculature assessed for physiological and pathogical changes. Mitochondrial respiratory activity was then assessed in wild-type and knockout animals vessels and isolated cells for their reliance on oxidative and glycolytic metabolism. During atherogenesis, we find that Pink1 knockout affects development of plaque quality rather than plaque quantity by decreasing VSMC and extracellular matrix components, collagen and elastin. Pink1 protein is important in the wild-type VSMC response to metabolic stress and induced a compensatory increase in hexokinase II, which catalyses the first irreversible step in glycolysis. Pink1 appears to play an important role in VSMC energetics during atherogenesis but may also provide insight into the understanding of mitochondrial energetics in other diseases where the regulation of energetic switching between oxidative and glycolytic metabolism is found to be important.

## 1. Introduction

DNA damage and mitochondrial dysfunction are discerning characteristics of human atherosclerosis [1,2,3]. Regulation of mitochondrial integrity is controlled by the phosphatase and tensin homolog (PTEN)-induced kinase 1 (Pink1). Human plaque vascular smooth muscle cells (pVSMCs) age faster than surrounding vessel wall cells^11^ and consequentially rely on a p53-dependent cell signalling program that drives cyclin-dependent kinase inhibitor (CDKi) activity that sensitises plaque cells to readily undergo apoptosis [4,5]. While apoptosis may limit early VSMC hyperplasia accompanying atherogensis, the loss of proliferation is a characteristic of end-stage disease and is more characteristic of cap thinning and propensity for plaque rupture [2,6,7]. Work has shown that excess nuclear DNA damage and decreased repair capacity drive mitochondrial dysfunction in plaque VSMCs. Analysis of human plaque samples has shown that the cap is especially susceptible to DNA damage, with cells presenting significant loss of mitochondrial dependant oxidative phosphorylation [8]. Not surprisingly, the VSMC phenotype in the plaque is tightly coupled to the energetic state of the cell, with changes in VSMC plasticity directly related to changes in cellular metabolism [9]. This accelerated aging phenotype is also found in explant human VSMCs in culture [10]; for example, we found that cells isolated from human carotid endarterectomy specimens show loss of mitochondrial integrity as flagged by Pink1 [8,11].

Pink1 is a 63 kDa serine/threonine protein kinase that is constitutively expressed and shuttled to the mitochondria. When the respiratory chain fails, the mitochondrial membrane potential (mΔΨ) depolarises, and Pink1 is stabilised through phosphorylation and recruits Parkin to coordinate mitochondrial proteasomal degradation [8]. A fraction of Pink1 is held with hexokinase II at the mitochondrial membrane termed, mitoHKII. It binds voltage-dependent anion channel 1 (VDAC1) which interacts with adenosine nucleotide translocase (ANT) to provide a coupling between glycolysis and oxidative phosphorylation which appears to confer a survival advantage [12]. We hypothesised that as plaque VSMCs age, they accelerate mitochondrial dysfunction, which mediates an energetic switch that favours a compensatory glycolysis program to maintain energy homeostasis.

During this transition period, we find that human cells have an increased abundance in phosphorylation of the energy sensor Adenosine Triphosphate (ATP) sensor AMP Kinase (AMPK) at Thr172 and increased glycolytic protein expression in the plaque cap [8]. Importantly, AMPK also upregulates total hexokinase II (HK-II), which has been shown to provide cardioprotection against ischemia and offer an adaptive role in cell surivival [13]. Here, we genetically modified mice to exhibit features of mitochondrial dysfunction and accelerated vascular aging using a Pink1 smooth muscle cell-specific and inducible Cre/LoxP system to provide spatial and temporal control over Pink1 knockout.

We targeted Pink1 as it plays a central role in mitochondrial homeostasis but may also be important in the emerging role of mitochondrial dysfunction in atherosclerosis. Pink1 is nuclear encoded but becomes mitochondrial resident to protect vascular cells from mitochondrial stress and is essential for recycling of energetically failed mitochondrial organelles through autophagy [11] and we suggest that it indirectly protects plaque stability by maintaining a healthy pool of VSMCs [14]. Here, we aim to test: (1) What effect does Pink1 knockout have on plaque development, and is this purely on plaque quantity or plaque quality? (2) Is Pink1 essential for normal oxidative phosphorylation during atherogenesis?

## 2. Results

### 2.1. Pink1 Mice Generation

**Figure 1:**
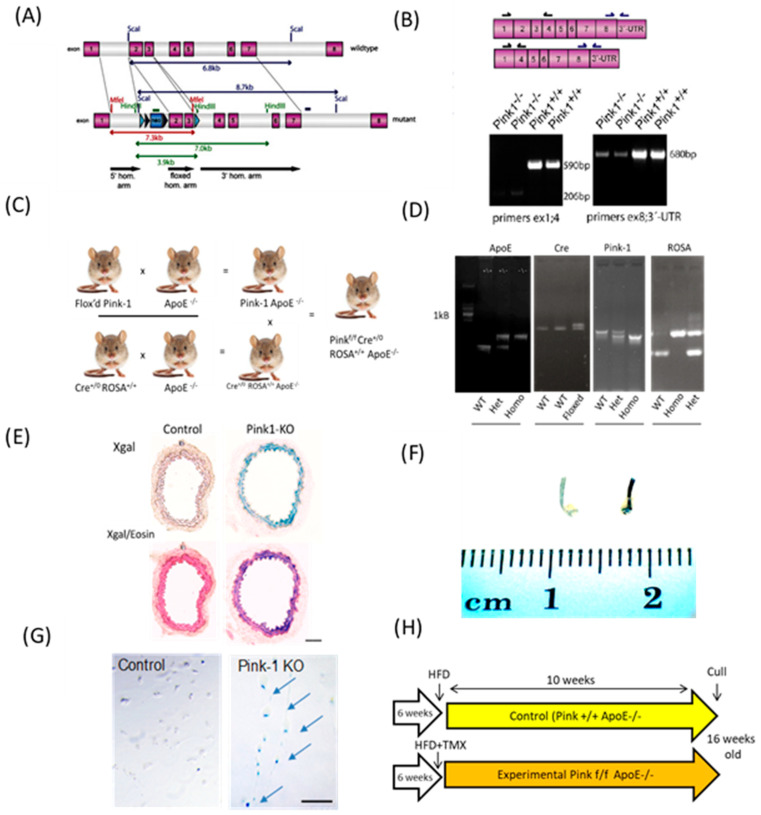
Mice breeding scheme. (**A**) Original Pink1^f/f^ knockout out mice locus and (**B**) truncation site and original Southern blot by Glasl et al. [15]. (**C**) Flox’d Pink1^f/f^ mice were crossed with ApoE^−/−^ animals. Double homozygotes were then bred with Cre^+/0^ Rosa^+/+^ ApoE^−/−^ mice. (**D**) DNA genotyping for each allele (**E**) Pink excision pilot study used a Cre/ROSA beta galactosidase reporter after IP injection with tamoxifen in corn oil (n = 3). Cre-recombinase and X-gal staining was confirmed across entire sections of aortic rings (upper pair) of vessels and is counterstained with eosin (lower pair). (**F**) Whole abdominal aortic tissue were stained blue in presence of X-gal when Pink was excised and Cre was activated. (**G**) A Pink1-KO VSMC line was derived from Pink^f/f^ explant whole aortae and treatedwith hydroxytamoxifen (OHT) in culture to mediate Pink1 excision using a beta-galactosidase reporter (arrows) (×10) (scale bar 100 µm). (**H**) The atherosclerosis feeding protocol included littermate and sex-matched controls with groups fed up to 16 weeks of age to generate atherosclerotic lesions.

The original Pink1^f/f^ knockout mice were gifted from Glasl et al. with the truncation site occurring between exons 1 and 4 and exon 8 and the 3′-UTR (Figure 1A), which was confirmed by them by Southern blot (Figure 1B) [15]. Flox’d Pink1^-f/f^ mice were crossed with ApoE^−/−^ animals (Figure 1C). Double homozygotes were then bred with Cre^+/0^ Rosa^+/+^ ApoE^−/−^ mice, with DNA genotyping completed for each allele (Figure 1D). A Pink excision pilot study used a Cre/ROSA beta galactosidase reporter after IP injection with tamoxifen in corn oil (n = 3). Cre-recombinase and X-gal staining was confirmed across entire sections of aortic rings (upper pair) of vessels and is counterstained with eosin (lower pair) (Figure 1E). Whole abdominal aortic tissue were stained blue in presence of X-gal when Pink was excised when Cre was activated. (Figure 1F). A Pink1-KO VSMC line was derived by explanting whole aortae and treating with hydroxytamoxifen (OHT) in culture (×10) (scale bar 100 µm) (Figure 1G). The atherosclerosis feeding protocol used littermate and sex-matched controls and the Pink1 transgenic group fed up to 16 weeks of age (Figure 1H).

### 2.2. Pink1 Mice Characterisation during High-Fat Feeding

**Figure 2:**
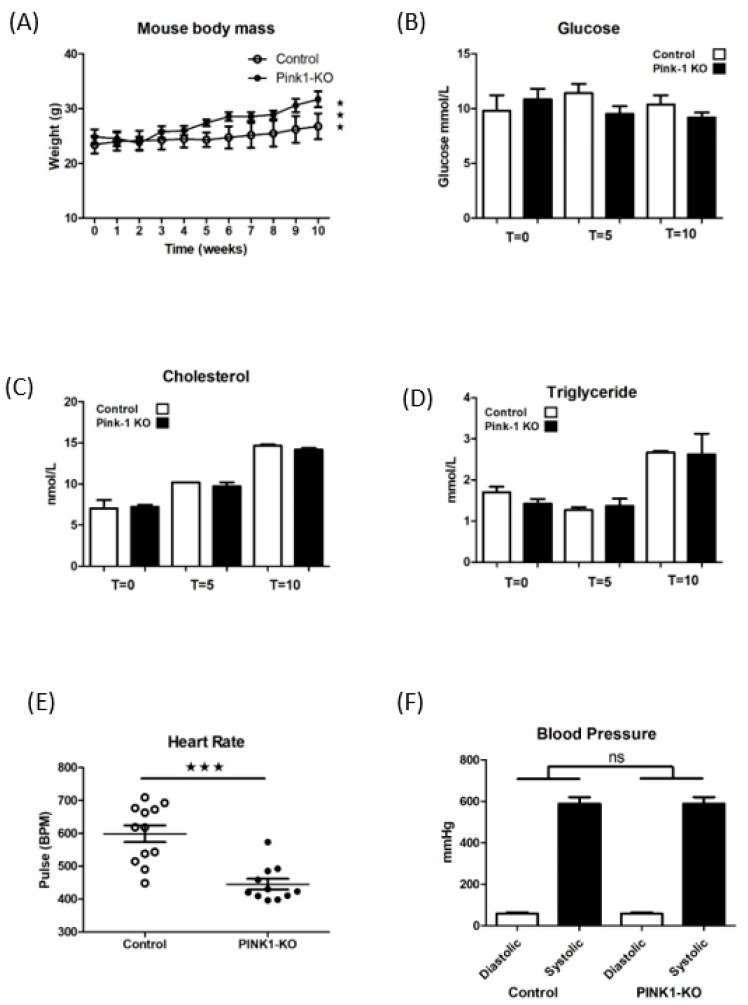
Pink1 mice body weight, serums and heart and blood pressures during high-fat feeding. (**A**) Body weights over experimental time course. (**B**) Blood serum values for glucose (**C**) cholesterol and (**D**) triglyceride across the high-fat feeding time course at T = 0, T = 5 and T = 10 weeks. (**E**) Mice heart rates @ T = 5 weeks. (**F**) Tail-cuff blood pressure values 5 weeks high-fat diet. Student *t*-test (n = 6) (ns—non significant), *** *p* ≤ 0.001. vs. control.

Mice were weaned at 4–6 weeks of age from a standard chow to a high-fat diet (See Section 4.2) and assessed for a number of physiological parameters and showed difference in weight (Figure 2A) but no change in serum glucose (Figure 2B) cholesterol or triglyceride. Readings were taken after 4 hr fast by tail bleed at 0, 5 and 10 weeks (on diet) and read immediately using a 3-in-1 handheld monitor (Prima^TM^). Indeed serum lipids rapidly increased across the feeding time course but there was no difference between any of the groups either at study start or by the end (Figure 2C,D). Mice had a significant difference in heart rate, (Figure 2E) but there was no significant difference in either diastolic or systolic blood pressures (Figure 2F).

### 2.3. Plaque Composition in Pink1-KO Mice

**Figure 3:**
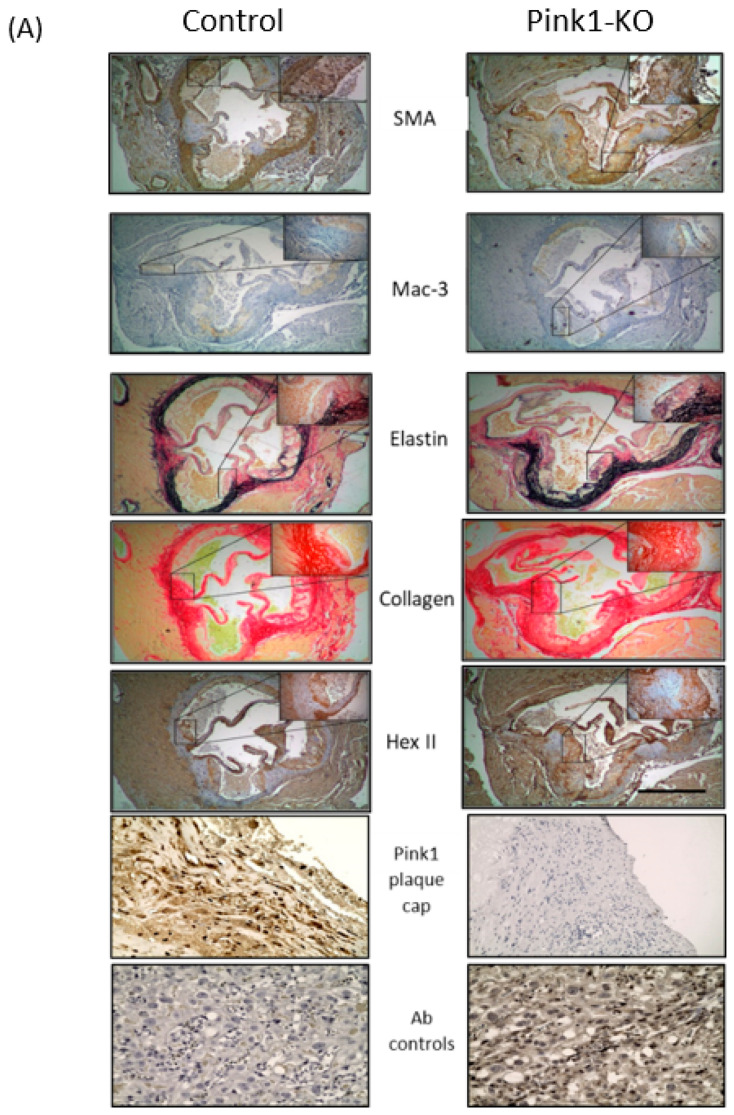

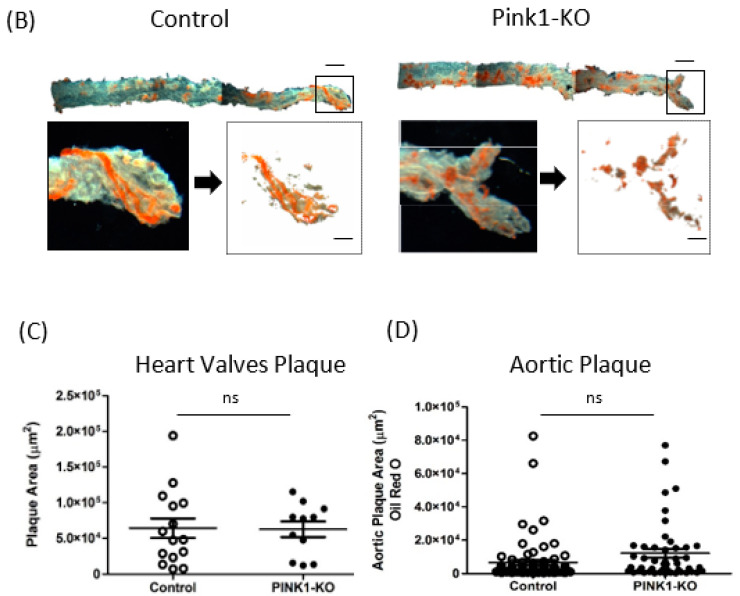
Pink1-KO ApoE^−/−^ mice have features of increased plaque vulnerability. Heart aortic root valves were sectioned at the coronary outflows and assessed for plaque quantity and quality. (**A**) Gross plaque structure and abundance of VSMC and macrophages, elastin andcollagens, hexII and Pink1 expression in plaque cap and IgG2A isotype controls (Olympus CellSenS) (scale 2 mm), control n = 9, Pink1-KO n = 15. (**B**) Representative whole aortae were taken from the aortic arch to the femoral bifurication and stained for total plaque burden with Oil red O, *en face* preparation (scale bar 500 µm). (**C**) Heart aortic valve (sinus) plaque cross-sectional area then quantified between groups. (**D**) Frequency of all discrete luminal surface aortic plaques were digitally extracted and plotted between control and Pink1-KO mice (Student *t*-test *p* ≥ 0.05, ns—not significant).

However, after a 10 week high-fat diet feeding regime to induce atherosclerosis, Pink1-KO mice had significant changes to the quality of plaque composition. The coronary sinus was used as a geographical landmark between animals to aid sectioning reproducibility (Figure 3A). The whole aorta was used as a measure of global plaque burden. Aortae were harvested from the aortic arch to the femoral bifurcation and stained with Oil red O to visualise and quantify fatty plaque deposits as previously described [2,6,7] (Figure 3B). Plaque area in heart valve plaques were quantified for each leaflet, with an average of 3 plaque per sinus (Figure 3C). Numerous individual plaques were scored across the entire luminal surface of the aorta and plotted between groups (Figure 3D). The abundance and distribution of aortic plaques across the thoracic and abdomen aorta were similar in littermates with the majority of plaques less than 2 × 10^4^ µm^2^ in size.

### 2.4. Quantification of Plaque Phenotypes in Pink1 Knockdown Model

**Figure 4:**
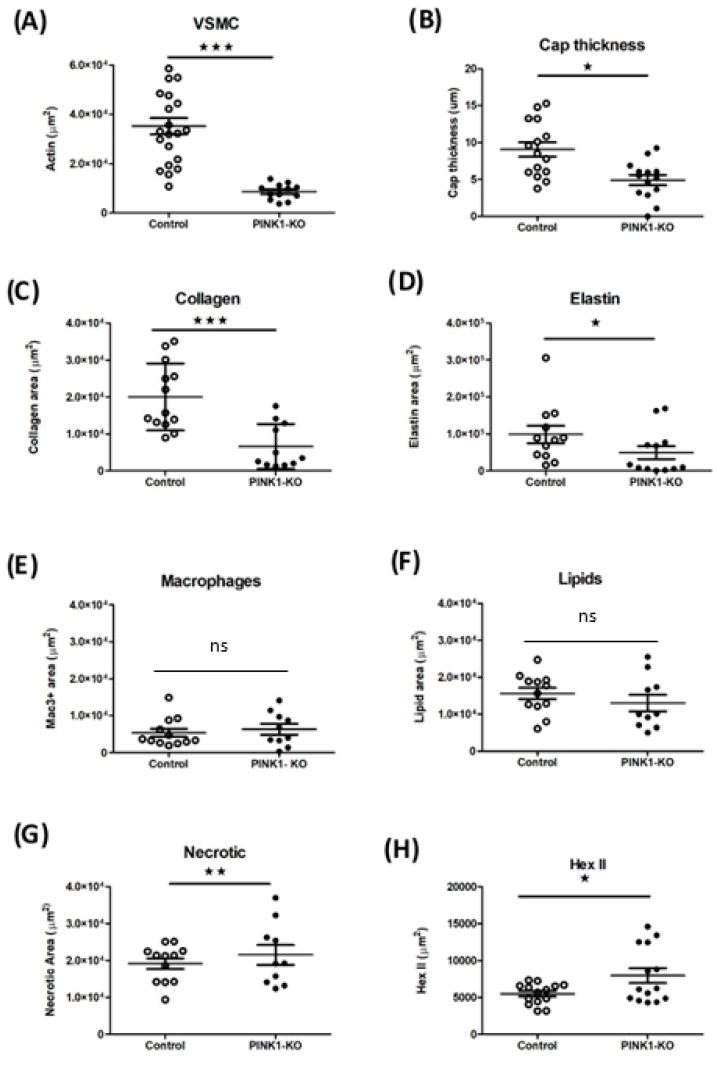
Pink1-KO ApoE^−/−^ mice have reduced VSMCs and extracellular plaque components. Quantification of plaque regions by immunohistochemistry for (**A**) VSMC (a-SMA+), (**B**) cap thickness, (**C**) extracellular collagen (sirius red), (**D**) elastin abundance (Verhoefff Van Gieson) stain, (**E**) macrophages (mac3+), (**F**) lipid void area, (**G**) necrotic area areas, and (**H**) hexokinase 2 abundance. Regions of stained plaque (n = 9–15). (ns—non significant), Student *t*-test. * *p* ≤ 0.05, ** *p* ≤ 0.01, *** *p* ≤ 0.001 vs. control.

Immunohistochemistry was quantified to evaluate plaque composition in the presence or absence of Pink1 (Table 1). Vascular smooth muscle actin-positive (SMA+) and monocyte/macrophage (Mac3+), collagen and elastin abundance were scored across each aortic sinus. A significant decrease in total SMA stained area for vessel wall (SMA+) (Figure 4A) was observed. This translated to a measurable decrease across the plaque cap thickness (Figure 4B). Picosirius red staining was used to estimate abundance of collagens, an important structural component of the plaque, with collagen type I accounting for over 90% collagen abundance in the plaque (Figure 4C). Verhoeff’s elastin van Gieson’s staining was used to estimate elastin and extracellular matrix deposition (Figure 4D). Significant changes in distribution of these markers were observed across the plaque commensurate with the reduction in the abundance of VSMC cells. In contrast, no change in tissue resident monocyte/macrophages (Mac3+) (Figure 4E), total lipid content (Figure 4F) and necrotic core area when scored as a composite of lipid void and foam cells regions was observed (Figure 4G). The calculation of a vulnerability index by the ratio of macrophage+lipids to the VSMC+collagen plaque component strongly supported increased vulnerability in Pink1 KO. An interesting observation is the abundance of hexokinase II protein, as a measure of glycolysis which appears limited to plaque cap and not medial VSMCs. This was notably increased in the plaque in the absence of Pink1 (Figure 4H).

### 2.5. In Vitro Studies

Having identified Pink1 as having a role in VSMC abundance in the plaque, we decided to characterise their energetic response *in vitro*. We tested their reliance of oxidative and glycolytic metabolism using the XF24 bioanalyser (Agilent, Santa Clara, CA, USA) for isolated cells and Oroborus^TM^ O2K for whole aortic vascular tissues. The activity of these pathways could then be used to predict if there was a direct effect on their energetic response and how this might correlate to their viability and synthetic capacity in the plaque.

**Figure 5:**
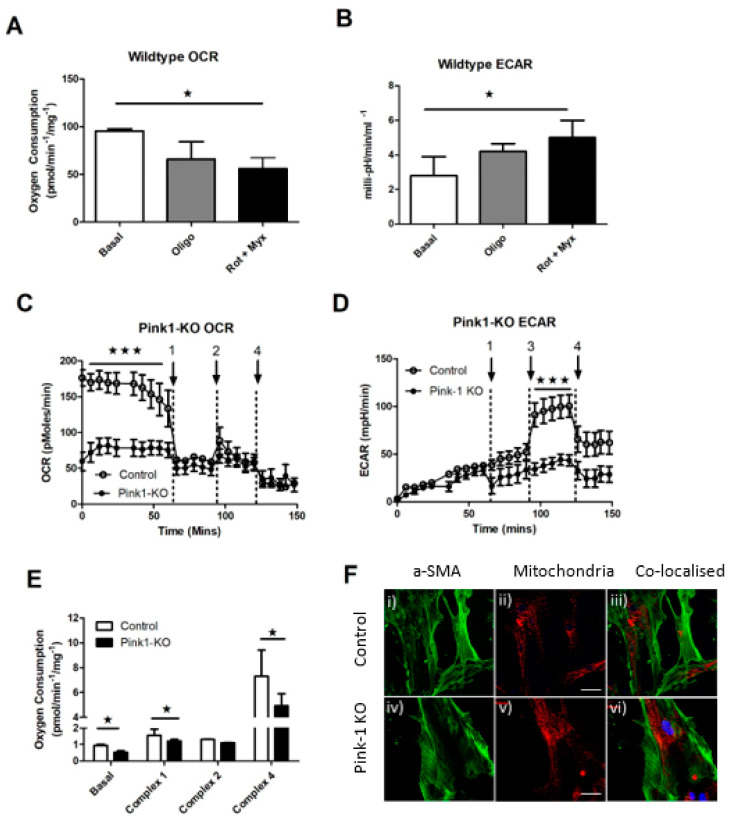
Pink1-KO ApoE^−/−^ mice have reduced respiratory capacity and ability to switch energetics to glycolysis compared to wild type. (**A**) Inhibition of mitochondrial respiration in Pink1+/+ wild-type VSMC retains a switching effect by (**B**) concomitant energetic switching by upregulation of glycolysis (ECAR). (**C**) In contrast, Pink1-KO cells have a decreased basal oxygen consumption rate (OCR) and sensitivity to inhibition of mitochondrial respiratory chain inhibitors; oligomycin (Arrow 1), uncoupler FCCP (Arrow 2) and rotenone and mxyothiazol (Arrow 4). (**D**) Loss of compensatory switch to glycolysis after oligomycin (Arrow 1) glucose bolus (Arrow 3) and 2-deoxy-glucose inhibitor 2-DG (Arrow 4) (n = 5). (**E**) Reduced mitochondrial oxygen consumption in Pink1 KO aortic tissue after high-fat diet both basally and specifically at complex I- and IV-dependent respiration. (**F**) Confocal images of mitochondrial VSMC α-SMA (green) (i–iv), mitochondrial abundance (red) (ii–v) with nuclear dapi (blue) co-localised combined channels to compare gross mitochondrial mass abundance between wild-type (Pink1+/+), control (upper) and Pink1 KO (lower) (n = 12) (scale 20 µm). One-way ANOVA with Dunnett’s post hoc test (n = 5), * *p* ≤ 0.05, *** *p* ≤ 0.001 vs. control.

To identify if Pink1 was essential for energetic switching, we first took wild-type cells (Pink+/+) only and assessed change in glycolysis as an increased extracellular acidification rate (ECAR) when the oxygen consumption rate (OCR) was inhibited. We took the basal unihibited rate as the control rate and compared any change relative to this internal control (Figure 5A,B). Using the Seahorse XF24 methodology allows this switch in the same cell line to be made at the same time for both parameters. We found that a reproducible switching effect was possible in wild-type VSMCs after titrating metabolic inhibitors oligomycin (complex V) and rotenone (complex I) and myxothaixzol (complex III). We found that when mitochondrial metabolism was suppressed by as little as 0.5 µM oligomycin, it resulted in ~25% suppression of respiration (Figure 5A) and this was sufficient for a commensurate switch and increase in glycolysis to occur (Figure 5B) and quantified in supplemental Table S1.

Then, using explant aortic VMSCs derived from control and Pink1-KO mice, we measured their rate of oxygen consumption (Oroboros Instruments, Innsbruk, Austria). In the absence of Pink1, there was a marked reduction in basal oxygen consumption (Figure 5C). Addition of 1 µM oligomycin inhibited ATP synthase (Arrow 1) and decoupled electron transport from ATP to be quantified. This rate plateaued in both cell lines creating a new baseline which remained unchanged after addition of the respiratory uncoupler carbonyl cyanide m-chlorophenyl hydrazone (CCCP, 1 µM) (Arrow 2) or rotenone, mxyothiazol (Arrow 4). Using the XF24 bioanalyser (Agilent), it was possible to simultaneously measure the glycolytic response in intact cells and in paired repeats (Figure 5D). Extracellular acidification (milli-pH) is a measure of glucose conversion of lactic acid as the end product of glycolysis which is pumped from the cytosol to the extracellular environment. We predicted that Pink1-KO would not be able to increase glycolysis when oxygen-dependent respiration was compromised by oligomycin (Arrow 1). Initially, both lines increased ECAR (milli-pH) during an initial equilibration phase over the first 50 min. Addition of a glucose bolus (Figure 5B—Arrow 3) drove a more robust wild-type glycolytic response over the next interval, in contrast to a slower and more delayed response from Pink1-KO cells, until all rates were inhibited with deoxyglucose and rotenone/myxothoazol (Arrow 4) (Supplemetal Table S1). This switching effect was then plotted relative to both OCR and ECAR (Supplemental Figure S2). To further isolate which part of the respiratory chain may be responsible *in vivo* for the Pink1-KO energetic response, we took fresh whole aorta from littermate control transgenic and knockout animals after 10 weeks high-fat diet (Figure 5E). Using control Pink1^+/+^ wild-type aorta, we ran paired samples with Pink1-KO mice aorta. Basal respiration was again compromised in Pink1-KO samples. We then proceeded to sequentially stimulate and inhibit each respiratory complex in turn. The residual rate provides a measure of the contribution of each complex to total respiration. This was achieved using the metabolic inhibitors oligomycin (complex V), rotenone and mxyothiazol (complex I/III) (Sigma Aldrich Ltd. Gillingham, UK) and tetramethyl-p-phenylenediamine (TMPD) as an artificial complex IV substrate until oxygen exhaustion occurred in the 02K chamber. This revealed a significant defect in complex I- and IV-dependent respiration.

## 3. Discussion

Loss of Pink1 during atherogenesis resulted in VSMC-specific changes in cell and extracellular matrix components independent of total plaque abundance. While the VSMC component of the vessel is high, their numeracy in the plaque is relatively low and so no change in total plaque quantity would be predicted, especially as loss of VSMC viability by itself does not drive plaque expansion. However, a reduction in VSMC content and collagen is critical to the mechanical strength of the plaque that ultimately drives plaque rupture, heart attacks and stroke. Though we did observe a decrease in Pink1 heart rate, this did not translate to a change in blood pressure. The mechanism for this is unclear but could relate to dynamic changes in mitochondrial function in both cardiac and vascular tissues. Nevertheless, these results, and the calculation of a vulnerability index, do suggest that Pink1 protects against atherosclerosis. This study links Pink1 to mitochondrial energetic dysfunction and plaque stability.

While histological analysis of plaque from aortic valves confirmed that loss of Pink1 decreased the VSMC population, there was no change in total plaque abundance or in the Mac-3+ inflammatory component of the plaque. The loss of extracellular matrix components and reduced abundance at such an early time point suggests that future studies should protract the high-fat feeding regime. Longer time points may have more significant effects to plaque structure and could be useful in modelling vulnerable plaque phenotypes found in some human lesions. Longer feeding regimes could also alter plaque remodelling and open the opportunity of testing plaque stabilising compounds. However, there are limitations in the number of histological sections possible at the aortic sinus and this limits quantification for other makers of interest such as Masons Trichrome and of low-abundance markers such as Ki67 for proliferation and cleaved caspase 3 for apoptosis. Indeed, measures of plaque cap thinning in combination with other histological measures may provide a particularly useful index in this model.

All cells will use a mixture of mitochondrial and glycolytic metabolism but as VSMCs age in the plaque as mitochondrial dysfunction increases. Previously, we found elevated Pink1 in the plaque cap of human carotid atherosclerosis samples [8]. Here, we find that Pink1 appears to be important in mediating the switch between mitochondrial respiration and glycolysis. Normally uncoupling respiration mimics an energetic dysfunction and forces the respiratory complexes to work at the maximal rate. The lack of response in both wild-type and Pink1-KO cells here suggests that respiration in these cells is already working at full capacity with no spare capacity when challenged. Rotenone inhibits complex I activity and mxyothiazol is a competitive inhibitor of ubiquinol at the bc1 complex and together these inhibitors block the transfer of electrons through iron sulphur complexes and their reduction to molecular oxygen, thereby inhibiting residual respiration. It is during this phase that we find that Pink1 appears important to allow the cells to switch to glycolysis. When then testing if these deficiencies induced in isolated cells occur *in vivo* during atherogenesis, we find respiratory chain defects in the first and last enzymes in the respiratory chain of fresh aortae. Triple staining VSMC α-SMC actin (green) nuclear counterstain (blue) co-localised to total mitochondria content using mitotraker red (Sigma Aldrich Ltd. Gillingham, UK) revealed no difference in VSMC mitochondrial content (Figure 5F) and or mitochodrial DNA copy number (Supplemental Figure S1 (i–iv)).

Hexokinase is a key enzyme associated with enhanced glycolysis and is increasingly recognised as part of the energtic survival nexus [16]. If the regulation of this switching effect could be targeted to the plaque via small molecules early in disease, than this has the potential to improve VSMC phenotype in the plaque. It could also help other cells and tissue in which mitochondrial energetics have been compromised. The fact that wild-type cells and tissues require Pink1 for an intact energetic switching response has led us to start screening for VSMC-specific compounds that can switch “on demand” from oxygen-depedent respiration to glycolysis and which may benefit VSMC longevity in the plaque.

Improved VSMC survival and modest increased proliferation would be predicted to strengthen the plaque cap and could theoretically delay rupture. The work of Juan Bolanos [17] suggests that significant rewiring of glycolytic homeostasis does occur during Pink1 ablation. However, these studies used mouse embryonic fibroblasts, (MEF’s) that relied on stem cell factors such as Lin28, which we have also shown to be an important mechanism in glycolytic switching [18]. We belive the terminally differentiated VSMCS used in our study to be more relevant to studying human disease.

In our study, we find that WT cells which retain Pink1 can switch energetics when oxphos is inhibited to then use glycolysis. In Pink1, KO oxygen respiration (OCR) is lower basally and fails to increase glycolysis (ECAR) when stimulated. Tentatively, we would suggest that Pink1 might tether a pool of mitoHexII to ensure a shift toward glycolysis can occur effectively when mitochondrial respiration is compromised

Indeed the work of Pan et al. [12] has shown that hexokinase 2 reduces calcium overload in coronary endothelial cells of type 2 diabetic mice and that may also mediate a beneficial effect here in vascular smooth muscle. Here, we explored how wild-type cells could be switched from dependence on mitochondrial energetics to glycolysis. In the future, it is hoped that wild-type VSMCs in the plaque could be pharmaceutically targeted. Indeed, designing appropriate targeting vehicles, such as tagged microvesicles that could be loaded with drug treatments during atherosclerosis is currently planned.

## 4. Materials and Methods

### 4.1. Generation of Pink1 Mice

Flox’d *Pink1* mice [15] were bred with standard *ApoE* homozygous mice from Jackson labs (B6.129P2ApoE^tm1Unc^/J—stock#002052). Mice homozygosis for both alleles (1:16) were genotyped and then crossed with *SMMHC-CreErT2* mice [18] from the University of Cambridge previously gifted by Professor Stefan Offermans (Max Planck Institute, Munich, Germany) under MTA to the University of Glasgow. These mice were previously crossed with a ROSA26 allele on the same C57/Blk/6 background. This created a unique Pink1 knockout mice at a ratio of 1:64 live births, termed here as knockout (*MHC-Cre^+/0^ ErT2 Pink1^f/f^ ROSA^+/+^ ApoE^−/−^).* To titrate tamoxifen dose and Cre leak, we performed a dose–response study by IP injection in Pink^f/f^ mice, including uninduced controls. All animal studies were approved by the UK Home office and met the University of Glasgow ethical review process.

### 4.2. Breeding and Feeding Regime

For breeding and husbandry, mice were fed a standard chow Cat#801002 (Special Diet Services, UK). This is a complete diet with 45% Starch, 17% fibre, 14% protein, 4% sugar supplemented with amino acids and micromineral and vitamins; see https://sdsdiets.com/wp-content/uploads/2021/02/rm1p-e-fg.pdf (Access date 8 September 2021). For atherogenesis studies, littermates were weaned at 4–6 weeks of age and transferred onto a “Western” high-fat diet (HFD) pellet supplied by Envigo, UK Cat#TD02028; see https://insights.envigo.com/hubfs/resources/data-sheets/02028.pdf (Access date 8 September 2021). For Pink1 excision, experimental animals were briefly fed a custom modified variant of TD02028 with 0.5 mg/g of tamoxifen base and red dye to distinguish from standard white HFD. Mice were then humanely culled and aortic vessels harvested at the study end at 16 weeks of age under an approved Home Office Licence.

### 4.3. Mice Characterisation

Mice were routinely weighed with blood pressures and heart rates taken using the tail-cuff method at 0, 5 and 10 weeks (Visitech System BP-2000 analyser). The transgenic mice genome also incorporated a Cre responsive flox’d STOP codon for the β-galactosidase reporter enzyme in the ROSA26 safe harbour locus. After genotyping with PCR and Taqman probes (Transnetyx, Cordova, TN, USA), we confirmed Cre activity and Pink1 knockdown in these mice by co-incubating aortic rings from frozen sections with X-gal substrate to provide a blue stain where gene knockout occurred across the entire vessel wall. These were then counterstained with haematoxylin and eosin. This method was also repeated in fresh explant abdominal aorta VSMCs, as the β-galactosidase enzyme retains activity both in frozen sections and after mild fixation [19]. A plaque vulnerability index as proposed by Bo Li et al. was calculated based on the of % macrophages + % lipids/% SMC + % Collagen [20].

### 4.4. Tissue Microdissection and Cell Sample Preparation

Whole aortic and heart tissue specimens were obtained after humane culling and rapid removal en bloc rather perfusion fixation as we found it difficult to maintain consistent plaque architecture. Fresh tissue samples were used for oxygen respirometry experiments and explant cell culture as previously described, while tissues for histology were post fixed in 4% paraformaldehyde for immunohistology or portions embedded in OCT compound for frozen sections for X-gal staining. Primary VSMCs were explant cultured in 10% serum supplemented with DMEM ( Sigma Aldrich Ltd., Gillingham, UK) and immunocytochemistry then used to determine lineage for myosin heavy chain (MHC), and α-smooth muscle cell actin (SMA) as previously described [7,10].

### 4.5. Generation of Pink1 Cell Lines

Pink1 excision was previously confirmed by Southern blotting, RT-PCR by Glasl et al. [15] and DNA genotyping and Western blot performed by ourselves [8] during breeding. Pink1-KO cell cells were routinuely generated from aortic explants as previously described and Pink1 KO *in vivo* confirmed by using the X-gal reporter in the ROSA26 locus. Briefly, tissues were harvested, washed in PBS and mounted in OCT compound and frozen sections cut at 5 µm intervals. For HFD animals in which tissues and cells were excised, these were treated with the metabolised form of tamoxifen, a low-dose hydroxytamoxifen (OHT), 100 nM, for a minimum of 48 hrs (Sigma Aldrich 68047-06-3) and then washed in 1 × PBS and fixed at room temperature for 5 min in 4% paraformaldehyde, supplemented with 0.1 N sodium PO_4_. Specimens were incubated overnight in 1 mL of staining buffer—200 mM MgCl_2_, 400 mM Ferricynanide, and 400 mM Ferrocynanide, X-gal solution in PBS—and then briefly washed in MQ and visualised.

### 4.6. Seahorse XF24 and Oxygraph Whole Tissue Oxygen Respirometry

Fresh mouse aortic tissues were permeabilised for 15 min with Saponin at 1:100 dilution of a 5 mg/mL solution of Saponin (Sigma Aldrich S4521). This was required for mitochondrial assays only, which disrupts the cholesterol rich plasma membrane and permits entry of substrates into the mitochondrial without losing integrity of the cholesterol poor mitochondrial outer membrane. Samples were further washed 3 × 5 min in BIOPS preservation solution (10 mM Ca-EGTA buffer, 0.1 µM free calcium, 20 mM imidazole, 20 mM taurine, 50 mM K-MES,0.5 mM dithiothreitol, 6.56 mM MgCl_2_, 5.77mM ATP, 15 mM phosphocreatine, pH 7.1) at 4 °C on a sample rotator at 25 rpm before paired tissue samples (~10 mg *w*/*w*) were added to the respirometry chambers (Oroboros Instruments, Innsbruck). Complex I respiration was energised with glutamate (10 mM) and malate (5 mM) and ADP before inhibition with rotenone (1 mM) to reveal a complex I-dependent oxygen consumption rate (OCR). Stimulation of complex II-dependent respiration was made by addition of the complex II substrate succinate (5 mM) and then inhibited by antimycin (5 mM). Complex IV respiration was stimulated with the electron donor ascorbate and artificial complex IV substrate TMPD (20 mM N,N,N′,N′-Tetramethyl-p-phenylenediaminedihydrochloride). A final addition of excess cytochrome c (4 mM) was used to show no further increase in respiration and confirmed the integrity of the respiratory chain preparation. All rates were normalised to dry tissue mass and immunohistology confirmed cell type and cell density determined for each region by eosin and haematoxylin (H&E) staining. The XF24 Seahorse assay was used intact cells for mitochondrial and glycolytic determination. Primary explanted murine VSMCs were seeded into microplate with 100 μL at 15,000 cells/well for normalistion. A calibration plate was prepared with 0.5 mL of XF calibrant solution. Cell plate medium was exchanged with 0% FCS Seahorse media on the day of the assay, with a minimum of five technical replicates per biological repeat. All injection ports were prepared as previously described [8].

### 4.7. Histology and Scoring Analysis

Histology and Oil red O staining were performed as previously described [2,6,7,20]. Goat anti-rabbit HRP secondary antibodies (A6154, Sigma) and Vector labs DAB secondary HRP staining kit (SK4100) for stain visualisation including secondary negative control and an infiltrating breast ductal carcinoma used a positive control (AMSBIO^TM^ CU2005/17). Images were captured and scored using an Olympus IX73 and BX51 microscopes using Adobe Photoshop^TM^ and Olympus CellSenS software. Plaque cap thickness was chosen by taking multiple measures at the region SMA+ that represented the plaque cap boundary and therefore the region with greatest vulnerability.

## Figures and Tables

**Table 1:** Atherosclerosis histology Pink1-KO ApoE^−/−^. Left columns atherosclerotic plaque areas SMA, Mac, cap thickness, collagen, elastin areas, lipid void area, necrotic area and areas with positive hexokinase 2-stained areas in control and Pink1-KO mice. Data are +/−SEM, * *p* ≤ 0.05, ** *p* ≤ 0.01, *** *p* ≤ 0.001 for control mice vs. Pink1-KO mice.

	Control (n = 6) Pink1^+/+^ApoE^−/−^	Experimental (n = 5) Pink1-KO ApoE^−/−^
**Aortic plaque area (%)**	12.59 +/−0.19	16.69 +/−0.99
	**Control (n = 15)** **Pink1^+/+^ApoE^−/−^**	**Experimental (n = 11)** **Pink1-KO ApoE^−/−^**
**Aortic root per plaque area (µm^2^) +/−SEM**	155,109 +/−81,716	124,191 +/−15,684
**SMA-positive area (µm^2^) +/−SEM**	35,168.48 +/−3333.19	8562.45 +/−1014.09 ***
**Cap thickness (µm) +/−SEM**	9.06 +/−1.04	5.29 +/−0.68
**Collagen positive (µm^2^) +/−SEM**	20,024.56 +/−2519.33	6646.79 +/−1744.31 ***
**Elastin-positive area (µm^2^) +/−SEM**	98,687.32 +/−23,026.61	49,638.60 +/−17,719.22 *
**MAC-positive area (µm^2^) +/−SEM**	5377.96 +/−1108.07	6326.55 +/−1436.95
**Lipid area (µm^2^) +/−SEM**	15,567.87 +/−1558.28	12,989.68 +/−2256.87
**Necrotic area (µm^2^) +/−SEM**	19,150.21 +/−1449.72	21,470.12 +/−2669.77 **
**Hexokinase 2 area (µm^2^) +/−SEM**	5500.82 +/−379.00	7953.43 +/−1005.76 *

## Data Availability

Access to datasets are available upon request.

## Supplementary Materials

The following are available online at https://www.mdpi.com/article/10.3390/ijms22189993/s1.

## References

[1] Gray K., Kumar S., Figg N., Harrison J., Baker L., Mercer J., Littlewood T., Bennett M. (2015). Effects of DNA damage in smooth muscle cells in atherosclerosis. Circ. Res..

[2] Mercer J.R., Cheng K.K., Figg N., Gorenne I., Mahmoudi M., Griffin J., Vidal-Puig A., Logan A., Murphy M.P., Bennett M. (2010). DNA damage links mitochondrial dysfunction to atherosclerosis and the metabolic syndrome. Circ. Res..

[3] Nahapetyan H.M.M., Grousset E., Faccini J., Grazide M.H., Mucher E., Elbaz M., Martinet W., Vindis C. (2019). Altered mitochondrial quality control in Atg7-deficient VSMCs promotes enhanced apoptosis and is linked to unstable atherosclerotic plaque phenotype. Cell Death Dis..

[4] Bennett M.R.S., Owens G.K. (2016). Vascular Smooth Muscle Cells in Atherosclerosis. Circ. Res..

[5] Bennett M.R., Littlewood T.D., Schwartz S.M., Weissberg P.L. (1997). Increased sensitivity of human vascular smooth muscle cells from atherosclerotic plaques to p53-mediated apoptosis. Circ. Res..

[6] Mercer J.R., Yu E., Figg N., Cheng K.K., Prime T.A., Griffin J.L., Masoodi M., Vidal-Puig A., Murphy M.P., Bennett M.R. (2012). The mitochondria-targeted antioxidant MitoQ decreases features of the metabolic syndrome in ATM+/-/ApoE-/-mice. Free Radic. Biol. Med..

[7] Mercer J., Figg N., Stoneman V., Braganza D., Bennett M.R. (2005). Endogenous p53 protects vascular smooth muscle cells from apoptosis and reduces atherosclerosis in ApoE knockout mice. Circ. Res..

[8] Docherty C.K., Carswell A., Friel E., Mercer J.R. (2018). Impaired mitochondrial respiration in human carotid plaque atherosclerosis: A potential role for Pink1 in vascular smooth muscle cell energetics. Atherosclerosis.

[9] Salabei J.K., Hill B.G. (2013). Mitochondrial fission induced by platelet-derived growth factor regulates vascular smooth muscle cell bioenergetics and cell proliferation. Redox Biol..

[10] Matthews C., Gorenne I., Scott S., Figg N., Kirkpatrick P., Ritchie A., Goddard M., Bennett M. (2006). Vascular smooth muscle cells undergo telomere-based senescence in human atherosclerosis: Effects of telomerase and oxidative stress. Circ. Res..

[11] He L., Zhou Q., Huang Z., Xu J., Zhou H., Lv D., Lu L., Huang S., Tang M., Zhong J. (2019). PINK1/Parkin-mediated mitophagy promotes apelin-13-induced vascular smooth muscle cell proliferation by AMPKalpha and exacerbates atherosclerotic lesions. J. Cell. Physiol..

[12] Pan M., Han Y., Basu A., Dai A., Si R., Willson C., Balistrieri A., Scott B.T., Makino A. (2018). Overexpression of hexokinase 2 reduces mitochondrial calcium overload in coronary endothelial cells of type 2 diabetic mice. Am. J. Physiol. Cell Physiol..

[13] Tan V.P., Smith J.M., Tu M., Yu J.D., Ding E.Y., Miyamoto S. (2019). Dissociation of mitochondrial HK-II elicits mitophagy and confers cardioprotection against ischemia. Cell Death Dis..

[14] Grootaert M.O.J., Roth L., Schrijvers D.M., De Meyer G.R.Y., Martinet W. (2018). Defective Autophagy in Atherosclerosis: To Die or to Senesce?. Oxid. Med. Cell. Longev..

[15] Glasl L., Kloos K., Giesert F., Roethig A., Di Benedetto B., Kuhn R., Zhang J., Hafen U., Zerle J., Hofmann A. (2012). Pink1-deficiency in mice impairs gait, olfaction and serotonergic innervation of the olfactory bulb. Exp. Neurol..

[16] Roberts D., Miyamoto S. (2015). Hexokinase II integrates energy metabolism and cellular protection: Akting on mitochondria and TORCing to autophagy. Cell Death Differ..

[17] Requejo-Aguilar R., Lopez-Fabuel I., Fernandez E., Martins L.M., Almeida A., Bolanos J.P. (2014). PINK1 deficiency sustains cell proliferation by reprogramming glucose metabolism through HIF1. Nat. Commun..

[18] Docherty C.K., Salt I.P., Mercer J.R. (2016). Lin28A induces energetic switching to glycolytic metabolism in human embryonic kidney cells. Stem Cell Res. Ther..

[19] Takahashi M., Hakamata Y., Takeuchi K., Kobayashi E. (2003). Effects of different fixatives on beta-galactosidase activity. J. Histochem. Cytochem..

[20] Li B., Zhao Y., Liu H., Meng B., Wang J., Qi T., Zhang H., Li T., Zhao P., Sun H. (2016). Visfatin Destabilizes Atherosclerotic Plaques in Apolipoprotein E–Deficient Mice. PLoS ONE.

