# Supplementary material for: An Inducible and Vascular Smooth Muscle Cell-Specific Pink1 Knockout Induces Mitochondrial Energetic Dysfunction during Atherogenesis
Source: Int J Mol Sci. 2021 Sep 16;22(18):9993. doi: 10.3390/ijms22189993 (PMC8467198; doi:10.3390/ijms22189993)
Supplement: Supplementary file 1 [file ijms-22-09993-s001.zip › ijms-1360530-supplementary.pdf]

Supplemental Figures

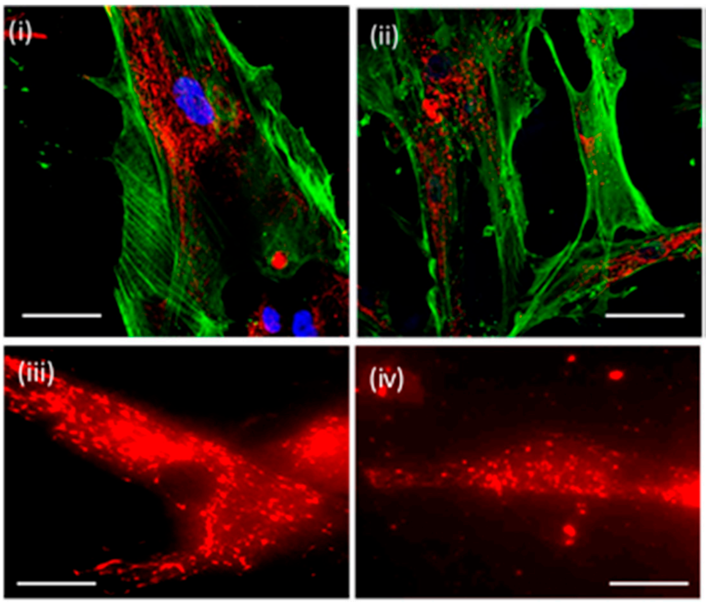

Figure S1. High resolution confocal images of mitochondrial abundance in **i)** Control Pink+/+ VSMC (green FITC) Mitotraker red (CMXRos) and nuclear dapi (blue) and ii) Pink1-KO VSMC and mitotracker red only in control (iii) and Pink1-KO (iv). (Scale 50 μm).

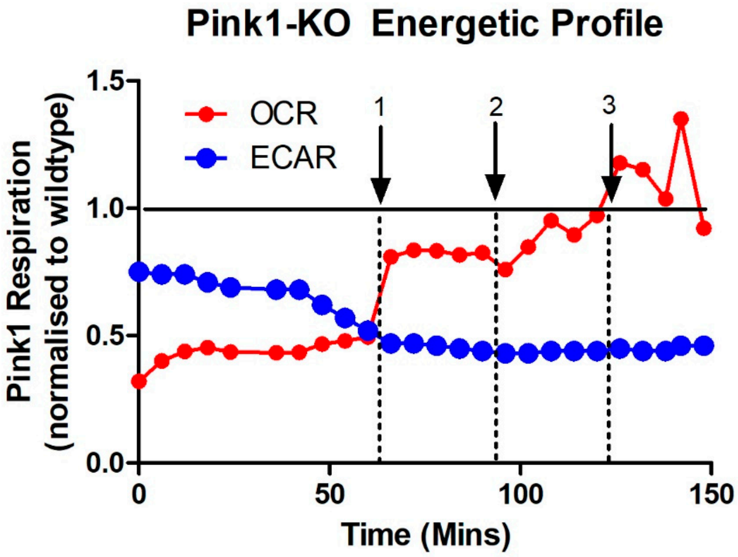

Figure S2. Normalised of OCR and ECAR data to wildtype control confirms Pink1 energetic profile remain below control values throughout the different phases of 1. basal,2. induction and 3.inhibition for both energy pathways until end phase.

Table S1. Quantification of energetic flux data between wildtype and Pink1-KO aortic VSMCs. Includes basal oxygen respiration rate, rate after oligomycin termed Proton leak, the difference between these as ATP production, and uncoupled Maximal respiration (n=5). For glycolysis; the basal rate, glycolytic rate induced after oligomycin, maximal glycolytic capacity after glucose bolus and the difference (p=001 between the two as glycolytic reserve before inhibition by 2DG (n=5). *** p=<0.001 paired t-test (n=5).

**Oxygen Consumption Rate (pmol/ml/min) (n=5)**	**Basal respiration**	**Proton Leak**	**ATP Production**	**Maximal Respiration**	**Spare Capacity**
Control Pink-1 WT	162.25 +/-5.25	62.11 +/-0.92	100.14 +/-5.92	68.83 +/-6.27	None
Experimental Pink1 KO	75.98 +/-3.31***	51.20 +/-3.67***	24.79 +/-4.20***	60.17 +/-3.65	None
**Extracellular Acidification Rate (milli-pH) (n=5)**	**Basal rate**	**Glucose induced**	**Glycolytic Capacity**	**Glycolytic Reserve**	**End rate**
Control Pink-1 WT	24.52 +/-6.71	46.37 +/-3.00	96.81 +/-5.07	72.28 +/-1.63	62.22 +/-5.14
Experimental Pink1 KO	18.47 +/- 1.86	26.52 +/-3.903***	40.09 +/-2.64***	21.62 +/-4.07***	27.90 +/-3.69
